# Adverse Events Following *Haemophilus influenzae* Type b (Hib) Monovalent Vaccines in Zhejiang Province, China, from 2017 to 2023

**DOI:** 10.3390/vaccines13040349

**Published:** 2025-03-25

**Authors:** Xuejiao Pan, Yaping Chen, Hui Liang, Linzhi Shen, Xiaohua Qi

**Affiliations:** Institute of Immunization and Prevention, Zhejiang Center for Disease Control and Prevention, Hangzhou 310051, China; xjpan@cdc.zj.cn (X.P.); ypchen@cdc.zj.cn (Y.C.); hliang@cdc.zj.cn (H.L.); lzhshen@cdc.zj.cn (L.S.)

**Keywords:** *Haemophilus influenzae* type b (Hib), adverse events, AEFI, surveillance, China

## Abstract

Background: The *Haemophilus influenzae* type b (Hib) vaccination has well-established safety and efficacy in preventing Hib and reducing related morbidity and mortality worldwide. China has a high disease burden of Hib yet a low coverage rate of Hib vaccines and remains the only WHO member country not including the Hib vaccine in its National Immunization Program (NIP), partly due to insufficient surveillance data on its safety. This study analyzed all adverse events following immunization (AEFIs) after Hib monovalent vaccination in Zhejiang Province from 2017 to 2023 to provide evidence for formulating relevant immunization strategies. Methods: Hib vaccine-related AEFIs in Zhejiang Province from 1 January 2017, to 31 December 2023, were collected through the Chinese National AEFI Information System (CNAEFIS) for a descriptive epidemiological analysis. Results: From 2017 to 2023, a total of 1740 Hib vaccine-related AEFIs were reported (incidence rate: 63.01/100,000 doses) (95%CI: 60.12/100,000 doses–66.04/100,000 doses), including 1577 common adverse reactions (57.10/100,000 doses) (95%CI: 54.35/100,000 doses–59.99/100,000 doses), 139 rare adverse reactions (5.03/100,000 doses) (95%CI: 4.26/100,000 doses–5.94/100,000 doses), and 24 coincidental events (0.87/100,000 doses) (95%CI: 0.58/100,000 doses–1.29/100,000 doses). Most of the AEFIs were common adverse reactions that manifested mainly as fever, injection site redness and swelling, and crying. AEFIs were more likely to occur in male participants under the age of one, in summer and autumn, and during the booster immunity stage. The top three regions with the highest reported incidence rates of AEFIs were Jiaxing City (86.61/100,000 doses) (95%CI: 74.44/100,000 doses–100.77/100,000 doses), Hangzhou City (81.29/100,000 doses) (95%CI: 72.56/100,000 doses–91.07/100,000 doses), and Taizhou City (66.62/100,000 doses) (95%CI: 58.24/100,000 doses–76.21/100,000 doses). Conclusions: Our findings provide preliminary evidence of the safety profile of the Hib vaccine at a provincial level, which adds further support for its broader implementation in other provinces. Future multi-center studies are needed to construct a comprehensive vaccine evaluation framework and make multi-criteria decisions on the feasibility of incorporating the Hib vaccine into China’s NIP.

## 1. Introduction

*Haemophilus influenzae* type b (Hib), a Gram-negative coccobacillus mostly affecting children’s respiratory tract, is a significant public health concern worldwide [1]. Hib is a highly pathogenic and contagious infectious disease mainly transmitted through airborne droplets from an infected person’s nasal secretions, mainly affecting children under 5 years old [2]. Hib can lead to a wide range of diseases, with meningitis and pneumonia being the two most common and deadly ones, contributing to the largest disease burden globally [1]. According to the World Health Organization (WHO), Hib accounts for over 90% of systemic infections caused by *Haemophilus influenzae*, leading to an annual 3 million serious cases each year [2].

Vaccination remains the only and most cost-effective health intervention to prevent Hib disease. It is available in a monovalent preparation (Hib monovalent vaccines) or in combination with other vaccines (Hib conjugate vaccines), with a recommended 3-dose scheme [2]. Hib conjugate vaccines are most commonly used worldwide, including PedvaxHIB, ActHIB, Hiberix, COMVAX, and Pentacel [2]. Hib conjugate vaccination offers long-term protective immunity against invasive Hib disease, with well-established safety and efficacy profiles. A meta-analysis of the dose-specific efficacy of the Hib conjugate vaccine showed that the pooled vaccine efficacy against invasive Hib disease was 59% for the one-dose scheme, 92% for the two-dose scheme, and 93% for the three-dose scheme [3]. In addition, Hib conjugate vaccines can help combat the global challenge of antimicrobial resistance (AMR) by reducing antibiotic use among children that would otherwise be used to treat Hib diseases [4]. Recognizing the significant social and health benefits of Hib conjugate vaccination, the WHO recommends that all member countries and regions should include Hib conjugate vaccines in their National Immunization Program (NIP) for all infants, regardless of surveillance data [5]. As of 2023, all 194 WHO member countries and regions, except China, have included the Hib conjugate vaccine in their NIP, and countries with high Hib conjugate vaccine coverage have essentially eliminated invasive Hib-related diseases [6].

In China, the Hib vaccine is not included in the NIP and can only be purchased out-of-pocket through the private market at a high price [7]. The average cost for the Hib monovalent vaccine ranges from RMB 50 to 100 (approximately USD 6.91–13.82), and the cost for the Hib conjugate vaccine is even higher, with the Pentacel costing around RMB 700 (approximately USD 96.71). Therefore, the Hib monovalent vaccine is most used in China, and the coverage rate remains low, with high inequalities between more and less developed areas in China [8]. Although China introduced the Hib monovalent vaccine in 1996, the coverage rate was only 42.6% (95%CI is 41.3%–44.0%) for the one-dose scheme and 25.0% (95%CI is 23.7%–26.3%) for the three-dose scheme in 2023 [8]. Additionally, regional differences exist across different provinces in China, with a coverage rate of 57.35% in Zhejiang Province [9], 53.53% in Anhui Province [10], and 22.77% in Jiangsu Province [11]. In China, at least half of the children do not enjoy the protection of Hib monovalent vaccines and are thus at a high risk of Hib infection and related morbidity and mortality. On the other hand, China has a high disease burden of Hib, accounting for 11% of global Hib deaths, despite a worldwide decreasing trend in the disease burden [8]. In 2015, approximately 78,000 children aged under five suffered from severe Hib disease, and nearly 3400 died from Hib infection in China [12]. In 2017, the national Hib infection mortality rate for 5-year-old children was 4/100,000, accounting for 1% of all deaths in China [6]. It is estimated that incorporating the Hib vaccine into China’s NIP can prevent approximately 235,700 Hib cases (93% reduction) and 2700 Hib deaths (92% reduction) in children under 5 years old every year while bringing about an increase of approximately 85,000 quality-adjusted life years (QALY) over the lifetime of this population [7].

Despite the high disease burden of Hib in China and the well-demonstrated cost-effectiveness of Hib conjugate vaccines worldwide, China remains the only country that does not include the Hib vaccine in its NIP, mainly due to a lack of high-quality evidence, especially relating to its safety profile [7]. Additionally, the international evidence is predominantly focused on the effectiveness and safety of Hib conjugate vaccines, and little research attention has been paid to the Hib monovalent vaccines that are more commonly adopted in China due to much lower prices. This research gap indicates the need for further research on the effectiveness and safety of the more affordable Hib monovalent vaccines in China to provide evidence to inform policymaking. The inclusion of Hib vaccines in China’s NIP has been listed as a policy priority to ensure universal vaccine coverage and prevent Hib-related mortality and morbidity [8]. In 2019, China issued the Vaccine Management Law, empowering each province to make its own policy regarding the introduction of Hib vaccines to its local immunization program [13]. Surveillance data at the provincial level is helpful, as it can provide further evidence and justification for the future inclusion of the Hib vaccine into the NIP [6]. As a coastal province in eastern China with relatively higher socioeconomic development, Zhejiang Province always has a high coverage rate of non-NPI vaccinations, including Hib vaccines. However, large-scale data on the safety profile of the Hib vaccine in Zhejiang Province have been lacking in recent years. This study analyzed all adverse events following immunization (AEFIs) after Hib monovalent vaccination in Zhejiang Province from 2017 to 2023. By evaluating its safety in a large-scale population over a long period, we aimed to provide an evidence basis to inform policy priorities to promote the inclusion of Hib vaccines into China’s NIP in the future.

## 2. Materials and Methods

### 2.1. Data Sources

Although vaccines must pass extensive safety tests and efficacy evaluations in laboratories, animals, and humans before they are launched on the market, adverse reactions that are rare or occur long after vaccination cannot be fully known during the trial phase [14]. Therefore, the WHO recommends that all countries should establish AEFI surveillance systems to continuously monitor vaccine safety and make timely adjustments to vaccination strategies, which can help optimize vaccination services and achieve a risk–benefit balance [15].

In 2008, China implemented its first nationwide, online AEFI surveillance system, named the Chinese National AEFI Information System (CNAEFIS), to collect adverse reactions or events that occur after vaccination and are suspected to be related to vaccination at a national level [16]. Details about the CNAEFIS and the surveillance of AEFIs have been described previously [16]. Briefly, the CNAEFIS is managed by the Chinese Center for Disease Control and Prevention (CDC) and covers all parts of the country at all levels. AEFIs are reported either directly online or indirectly via mail by medical institutions, vaccination units, and other organizations or personnel with reporting responsibility [16]. A professional evaluation team of the CDC performed further investigations, diagnoses, and classifications, as well as conducted re-evaluations to resolve any disagreements or disputes.

In this study, Hib monovalent vaccine-related AEFIs in Zhejiang Province from 1 January 2017 to 31 December 2023 were collected through the CNAEFIS. Information on the vaccine administration and doses during the same period was collected through the Zhejiang Province Comprehensive Management Information System for Vaccines and Immunization.

### 2.2. Definition and Classification

AEFIs are defined as any reaction or event that occurs after vaccination and is suspected to be related to the vaccine, which can potentially cause organ or functional damage [17]. According to the causes, AEFIs can be categorized into six types: common adverse reactions, rare adverse reactions, coincidental events, vaccine quality accidents, vaccination accidents, and psychogenic reactions [16]. For this study, we only described the first three types due to a zero-reporting rate for the other types. Common adverse reactions are usually minor reactions that will only cause transient body dysfunction, such as swelling, induration, and fever. Rare adverse reactions could be severe reactions after a standard vaccination that damage the recipient’s organs and tissues, leading to significant dysfunctions. Coincidental events refer to a medical condition that is not caused by the vaccine but occurs after vaccination, simply coinciding with the timing of the vaccination. According to severity, AEFIs can be categorized into severe and non-severe cases. Severe AEFIs refer to any reaction that may endanger one’s life, require hospitalization or other medical interventions, and may lead to physical disability or even death. Non-severe cases refer to reactions that can usually be recovered from automatically without hospitalization or further treatment.

### 2.3. Data Collection

The Hib vaccine is not included in China’s NIP, and each province has its own vaccination plan. In Zhejiang Province, the Hib vaccination procedure is carried out according to the instructions, and the vaccination scheme varies according to the age of vaccine initiation. The recommended standard immunization schedule is the 3 + 1 scheme, which includes three primary doses injected at a 1–2-month interval beginning at 2–3 months old (the basic immunity stage), followed by a booster dose at 18 months old (the booster immunity stage). For infants whose Hib vaccination is initiated between 6 and 12 months old, the following 2 + 1 scheme is recommended: two primary doses injected at a 1–2-month interval, followed by a booster dose at 18 months old. Children aged 1 to 5 years old only need to be vaccinated with one primary dose. The vaccine must be shaken thoroughly until it is completely dissolved before inoculation. Intramuscular or deep subcutaneous injections should be selected, while intravenous injections are strictly prohibited. The recommended injection site is the anterolateral thigh (middle 1/3) for infants and young children and the deltoid area of the upper arm for older children.

This study collected all the Hib cases that reported AEFIs in Zhejiang Province from 2017 to 2023. The Hib vaccine can be administered with other injectable vaccines at the same time (such as the diphtheria–tetanus–pertussis [DTP] vaccine, measles–mumps vaccine, etc.). Therefore, we excluded AEFIs that were caused by other vaccines administered concomitantly, which usually presented with only local reactions at the injection site, such as redness, swelling, induration, etc., without other systemic symptoms. For example, if the Hib vaccine (left upper arm) and DTP vaccine (right upper arm) were administered at the same time, and local induration occurred in the right upper arm, but without other systemic symptoms, it would be considered an AEFI caused by the DTP vaccine and excluded from the analysis.

Ethical approval was obtained from the Ethics and Research Committees of Zhejiang CDC (2025-002-01, approval date: 21 January 2025). Due to the study’s observational nature and the use of a de-identified dataset, informed consent was waived by the Ethics Committee.

### 2.4. Statistical Analysis

The data were analyzed utilizing R4. 4.1 software. Descriptive statistics were used to describe the incidence and characteristics of Hib-related AEFIs. The incidence rate (/1 million) was calculated as the number of AEFI reports/administered doses × 1 million. We further compared the incidence rates by different characteristics using the Cochran–Armitage trend test, with Bonferroni correction for pairwise comparisons and a *p*-value of < 0.05 indicating a statistically significant difference.

## 3. Results

### 3.1. Overview of Hib Vaccine-Related AEFIs

Table 1 shows the reported Hib vaccine-related AEFIs by year and category. A total of 2.7616 million doses of Hib vaccine were administered in Zhejiang Province from 2017 to 2023, and the number of doses decreased sharply by 71.91%, from 613,600 in 2017 to 172,300 in 2023. During the same time, a total of 1740 Hib-related AEFI cases were reported (incidence rate: 63.01/100,000 doses) (95%CI: 60.12/100,000 doses–66.04/100,000 doses). From 2017 to 2023, the reported incidence rate varied annually, ranging from 48.24/100,000 doses to 83.30/100,000 doses, yet with no statistical significance (*Z* = 0.0409, *p* = 0.9674). For the occurrence time, among the 1740 AEFI cases, 1353 (77.76%) were reported within the same day of vaccination, 366 (21.03%) were reported within 1–3 days after vaccination, and the remaining 21 cases (1.21%) were reported more than 4 days after vaccination. The longest interval between vaccination and AEFIs was 36 days.

**Figure 1:**
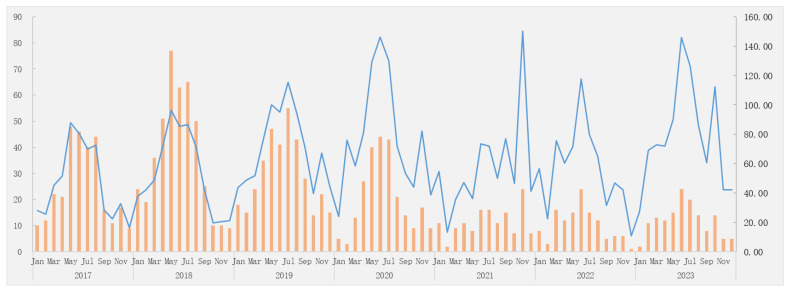
The reporting trends of AEFIs following Hib vaccination from 2017 to 2023 (/100,000 doses). Legend: column, the number of AEFI cases; row, the reporting time (year and month); the orange represents the number of AEFI cases; the blue lines indicate the reported incidence rate of AEFI cases. Hib: *Haemophilus influenzae* type b; AEFIs: adverse events following immunization.

For the AEFI category, among the 1740 AEFI cases, 1577 cases (90.63%) were common adverse reactions, 139 (7.99%) were rare adverse reactions, and 24 (1.38%) were coincidental events, corresponding to an incidence rate of 57.10/100,000 doses (95%CI: 54.35–59.99), 5.03/100,000 doses (95%CI: 4.26–5.94), and 0.87/100,000 doses (95%CI: 0.58–1.29), respectively. In addition, six were severe cases (incidence rate: 0.22/100,000 doses) (95%CI: 0.10–0.47), while the rest were non-severe cases. Figure 1 graphically presents the changes in the reported Hib vaccine-related AEFIs over time from 2017 to 2023. The AEFIs showed a wave pattern with peaks occurring in the summer and winter seasons.

### 3.2. Clinical Symptoms

We identified 1577 cases of common adverse reactions, and Table 2 shows the distribution of these clinical symptoms. The three most frequently reported symptoms were fever (52.82%), injection site redness and swelling (39.31%), and crying (22.76%), with incidence rates being 33.28/100,000 doses (95%CI: 31.19/100,000 doses–35.50/100,000 doses), 24.77/100,000 doses (95%CI: 22.98/100,000 doses–26.69/100,000 doses), and 14.34/100,000 doses (95%CI: 12.99/100,000 doses–15.82/100,000 doses), respectively. Among the 919 fever cases, 488 had simple fever, and the rest were accompanied by one or more symptoms, such as redness, swelling, induration at the injection site, crying, lethargy, and a loss of appetite.

We also identified 139 cases of rare adverse reactions. As shown in Table 2, the most frequently reported symptom was allergic rash (*n* = 83; incidence rate: 3.01/100,000 doses) (95%CI: 2.42/100,000 doses–3.73/100,000 doses), followed by urticaria (*n* = 27; incidence rate: 0.98/100,000 doses) (95%CI: 0.67/100,000 doses–1.42/100,000 doses), and maculopapular rash (*n* = 14; incidence rate: 0.51/100,000 doses) (95%CI: 0.30/100,000 doses–0.85/100,000 doses). Other symptoms included angioedema, febrile convulsions, lymphadenitis and lymphangitis, thrombocytopenic purpura, acute allergic reactions and erythema multiforme, vomiting, convulsions, tics, etc.

### 3.3. Epidemiological Distribution

Table 3 shows the distribution of Hib vaccine-related AEFIs by gender, age, and season. Among the total 1740 cases, 993 were male, and 747 were female, with a male-to-female ratio of 1.33:1. Males had a significantly higher reported incidence rate of Hib vaccine-related AEFIs than females (68.94/100,000 vs. 56.53/100,000, *p* < 0.0001). Regarding age distribution, the reported incidence rates of AEFIs in the age groups, namely <1, 1, 2, 3, and ≥4 years, were 54.78/100,000 (95%CI: 51.13/100,000 doses–58.69/100,000 doses), 69.87/100,000 (95%CI: 65.11/100,000 doses–74.98/100,000 doses), 87.03/100,000 (95%CI: 72.79/100,000 doses–104.04/100,000 doses), 105.85/100,000 (95%CI: 74.16/100,000 doses–151.07/100,000 doses), and 65.36/100,000 (95%CI: 37.39/100,000 doses–114.22/100,000 doses), respectively, showing a first increasing and then decreasing trend with increasing age (*p* < 0.0001). Regarding seasonal distribution, the reported incidence rates of AEFIs in spring, summer, autumn, and winter were 43.92/100,000 (95%CI: 39.04/100,000 doses–49.42/100,000 doses), 86.13/100,000 (95%CI: 79.88/100,000 doses–92.86/100,000 doses), 73.51/100,000 (95%CI: 67.67/100,000 doses–79.85/100,000 doses), and 38.78/100,000 (95%CI: 34.05/100,000 doses–44.16/100,000 doses), respectively, with significant seasonal differences (*p* < 0.001).

**Figure 2:**
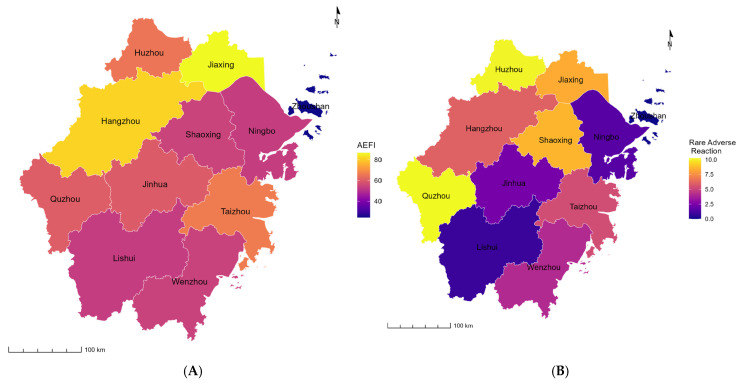
The incidence of Hib vaccine-related AEFIs by location from 2017 to 2023 in Zhejiang Province. AEFIs: adverse events following immunization. (**A**) AEFIs; (**B**) rare adverse reactions.

Table 4 and Figure 2 show the distribution of Hib vaccine-related AEFIs by location. The top three regions with the largest numbers of reported cases were Hangzhou City (n = 297), Wenzhou City (n = 253), and Jinhua City (n = 228). The top three regions with the highest reported incidence rates of AEFIs were Jiaxing City (86.61/100,000 doses) (95%CI: 74.44/100,000 doses–100.77/100,000 doses), Hangzhou City (81.29/100,000 doses) (95%CI: 72.56/100,000 doses–91.07/100,000 doses), and Taizhou City (66.62/100,000 doses) (95%CI: 58.24/100,000 doses–76.21/100,000 doses). Additionally, the top three regions with the highest reported incidence rates of rare adverse reactions were Quzhou City (10.23/100,000 doses) (95%CI: 5.85/100,000 doses–17.88/100,000 doses), Huzhou City (10.10/100,000 doses) (95%CI: 6.22/100,000 doses–16.41/100,000 doses), and Shaoxing (8.59/100,000 doses) (95%CI: 5.62/100,000 doses–13.14/100,000 doses).

### 3.4. Comparison of AEFIs by Doses

Table 5 shows the reported Hib vaccine-related AEFIs by different vaccination doses. The total numbers of AEFIs following the first, second, third, and fourth dose of the Hib vaccine were 732, 278, 285, and 445, respectively, corresponding to an incidence rate of 59.36/100,000 doses (95%CI: 55.21/100,000 doses–63.82/100,000 doses), 45.37/100,000 doses (95%CI: 40.35/100,000 doses–51.03/100,000 doses), 55.37/100,000 doses (95%CI: 49.31/100,000 doses–62.18/100,000 doses), and 110.94/100,000 doses (95%CI: 101.10/100,000 doses–121.73/100,000 doses), respectively, with statistically significant differences (*χ*^2^ = 184.0121, *p* < 0.0001).

We further compared each type of AEFI by different doses. Among the 1577 cases of common adverse reactions, 671, 250, 247, and 409 occurred after the first, second, third, and fourth doses. The reported incidence rates were 54.41/100,000 doses (95%CI: 50.45/100,000 doses–58.69/100,000 doses), 40.80/100,000 doses (95%CI: 36.05/100,000 doses–46.18/100,000 doses), 47.99/100,000 doses (95%CI: 42.37/100,000 doses–54.36/100,000 doses), and 101.96/100,000 doses (95%CI: 92.55/100,000 doses–112.33/100,000 doses), respectively, with statistically significant differences (*χ*^2^ = 179.0318, *p* < 0.0001). Among the 139 cases of rare adverse reactions, 49, 25, 36, and 29 occurred after the first, second, third, and fourth doses of the vaccination, respectively. Correspondingly, the incidence rates were 3.97/100,000 doses (95%CI: 3.01/100,000 doses–5.25/100,000 doses), 4.08/100,000 doses (95%CI: 2.76/100,000 doses–6.02/100,000 doses), 6.99/100,000 doses (95%CI: 5.05/100,000 doses–9.68/100,000 doses), and 7.23/100,000 doses (95%CI: 5.03/100,000 doses–10.38/100,000 doses), respectively, with statistically significant differences (*χ*^2^ = 11.6488, *p* = 0.0087).

### 3.5. Comparison of AEFIs by Co-Administration Status

Table 6 shows the comparison of Hib vaccine-related AEFIs by their co-administration status. Among the 1470 AEFIs, 1465 occurred following a single Hib vaccine, and the most common symptoms were fever (*n* = 72,349; 35%), local redness and swelling (*n* = 630; 43.00%), and crying (*n* = 323; 22.05%). In addition, 257 AEFIs occurred following co-administration with another vaccine (EV71 vaccine, inactivated polio vaccine, etc.), with common symptoms being fever (*n* = 185; 71.98%), local redness and swelling (*n* = 51; 19.84%), and crying (*n* = 67; 26.07%). Furthermore, 18 AEFIs occurred following co-administration with two other vaccines, with common symptoms being fever (*n* = 11; 61.11%), local redness and swelling (*n* = 3; 16.67%), and crying (*n* = 6; 33.33%).

The participants who either received or did not receive co-administration with other vaccines (one or two types) showed statistically significant differences in the reported incidence of fever (*χ*^2^ = 44.6467, *p* < 0.0001) and redness and swelling (*χ*^2^ = 52.9916, *p* < 0.0001), but no statistical difference in crying (*χ*^2^ = 2.6644, *p* = 0.1026).

### 3.6. Severe Cases and Outcomes

Among the total 1740 Hib-related AEFIs reported, 6 were classified as severe cases, with an incidence rate of 0.22/100,000 doses (95%CI: 0.10/100,000 doses–0.47/100,000 doses). Table 7 shows the characteristics of the six severe AEFIs, including the age, gender, vaccination time, vaccination doses, AEFI occurrence time, co-administration with other vaccines, causes, and clinical diagnosis. Among the six severe cases, five occurred after the first dose, and one occurred after the fourth dose, which was also co-administered with the fourth dose of the DPT vaccine. After investigation and diagnosis by the expert committee, five cases were classified as rare adverse reactions (incidence rate: 0.18/100,000 doses) (95%CI: 0.08/100,000 doses–0.42/100,000 doses), and the clinical diagnoses were febrile convulsions (three cases) and thrombocytopenic purpura (two cases). The remaining one case was classified as a coincidental event, as the patient had a history of allergic purpura for three months before receiving the Hib vaccine.

Among the 1740 AEFIs, 1654 cases had full recovery without treatment (95.06%), 78 cases recovered after treatment (4.48%), and the remaining 8 cases could not be tracked. No deaths or other severe sequelae were reported.

## 4. Discussion

Hib is a leading cause of mortality and morbidity among children aged under five worldwide, and the emergence of drug-resistant Hib has become an increasing global health concern due to the widespread use of antibiotics [1]. The “2030 Immunization Agenda” issued by the WHO and 25 other organizations mentioned that by 2030, the coverage rate of basic vaccinations in children and adolescents will reach 90%, and the number of children who are not vaccinated at all will be reduced by half [18]. As of 2023, 193 countries among the 194 WHO member states and regions have included the Hib vaccine in their NIP. China has a high disease burden of Hib yet remains the only country that does not include the Hib vaccine in its NIP, mainly due to a lack of high-quality evidence, especially relating to its safety profile [7]. Therefore, we conducted the current study to describe and analyze the incidence and characteristics of Hib vaccine-related AEFIs in Zhejiang Province from 2017 to 2023 to provide evidence of its safety at the provincial level and to inform further policymaking.

An interesting finding was that the number of Hib vaccinations in Zhejiang Province declined strikingly, by over 70%, from 2017 to 2023. This finding, although surprising, was consistent with a large body of previous studies showing that the Hib vaccination rate gradually decreases over time [19]. For instance, Chen et al. [20] found that the number of Hib vaccine doses in China dropped from 1567.1 million doses in 2014 to 6.829 million doses in 2020. This may be explained by the declining number of births over time and the decreased vaccinations during the COVID-19 pandemic [20]. Another possible reason may be that more parents choose Hib conjugate vaccines to decrease the vaccination doses. Compared to the Hib monovalent vaccines in our study, Hib conjugate vaccines have many advantages, including reducing the number of visits to vaccination clinics, decreasing children’s fear and discomfort, improving vaccination compliance, and enhancing the types of diseases covered. A Japanese study showed that Japanese mothers were more willing to vaccinate their children with combined vaccines to reduce clinic visits [21]. However, the relatively higher price of Hib conjugate vaccines has limited their wide use in China. There are currently a variety of Hib conjugate vaccines on the market, and our next research direction will be to compare their efficacy and safety.

Our study showed that the reported incidence rate of Hib vaccine-related AEFIs in Zhejiang Province from 2017 to 2023 was 63.01/100,000 doses, with no statistically significant differences between years. This rate was much higher than the national level of 38.10/100,000 doses from 2010 to 2021 [22]. When compared with other areas in China, this rate was similar to that of Beijing City from 2019 to 2020 (59.64/100,000 doses) [23] and to Jiaxing City and Zhejiang Province from 2016 to 2020 (66.06/100,000 doses) [24]. However, it was lower than that of Jiangsu Province from 2008 to 2014 (77.98/100,000 doses) [25], Suzhou City from 2014 to 2017 (97.3/100,000 doses) [26], and Shanghai City from 2007 to 2010 (132.73/100,000 doses) [27] but higher than that of Henan Province from 2011 to 2016 (10.55/100,000 doses) [28]. Noteworthily, the eastern areas account for the largest proportion of the national AEFIs, which may be explained by the high volume of vaccinations due to the large population, the high sensitivity in surveillance due to the extensive network and strengthened training, and the high level of AEFI awareness among the parents.

When compared with other countries where Hib conjugate vaccines are already included in NIPs, our study showed that the Hib vaccine demonstrated a relatively high safety profile in China. An early randomized controlled trial in the US showed that 13–30% of Hib vaccine recipients developed AEFIs, such as pain, swelling, and erythema [29]. Recent data based on the national vaccine safety surveillance program in the US, the Vaccine Adverse Events Reporting System (VAERS), 1990–2013, documented 29,747 AEFI reports, among which, 5179 (17%) were serious, including 896 deaths [30]. Another prospective, observational, post-marketing safety surveillance study among 646 South Korean infants reported 194 AEFIs in 143 infants (22.1%) [31]. Data based on the Japanese Adverse Drug Event Report database (JADER) from 2004 to 2017 documented 6280 AEFIs, among which, the most frequently reported vaccines for children aged <10 was the Hib conjugate vaccine (19.2%) [32]. However, it should be noted that different studies varied in their AEFI definitions, assessment, data sources, surveillance systems, study designs, and observation periods, which may hinder further cross-country and cross-study comparisons.

Our study revealed that most Hib vaccine-related AEFIs were common adverse reactions, with the top three most common symptoms being fever (52.82%), injection site redness and swelling (39.31%), and crying (22.76%). This finding was consistent with the US VAERS data, which showed that the most frequently reported symptoms of Hib-related AEFIs were fever (30%), injection site erythema (11%), crying (11%), irritability (10%), and rash (9%) [30]. Similarly, another study in South Korea showed that the most frequent AEFIs following the Hib vaccine were pyrexia (13.3%), injection site swelling (5.1%), and irritability (1.7%) [31]. A small portion of AEFIs were rare adverse reactions, mainly manifested as allergic rashes, followed by urticaria and maculopapular rash. In addition, most AEFIs showed favorable outcomes and resolved spontaneously without treatment. Only a few severe cases recovered after timely and reasonable treatment, with no sequelae or deaths reported. All these reactions were in line with the vaccine instructions, which clearly list a range of common adverse reactions observed during the clinical trial phase covering both local reactions (e.g., erythema, swelling, inflammation, and induration of the injection site) and systematic reactions (e.g., loss of appetite, irritability, lethargy, fever, vomiting, and crying). Our findings suggest that Hib vaccination is relatively safe to use in a large-scale population in Zhejiang Province. Still, it is necessary to strengthen the training of grassroots staff to improve their abilities in correctly recognizing AEFIs and appropriately managing them. Whenever they receive consultations and reports from the parents of vaccine recipients, they should comprehensively evaluate the reactions and provide reasonable treatment to reduce parent anxiety and avoid condition aggravation being caused by improper treatment.

A comparison of Hib vaccine-related AEFIs by gender, age, season, and region showed similar patterns to those in previous studies. Regarding gender, males had a higher incidence rate than females, with a male-to-female ratio of 1.33:1, which was similar to the reported 1.51:1 in Jiangsu Province [25] and 1.49:1 in Henan Province [28]. There is no scientific evidence to suggest that boys are more likely to have adverse reactions to the Hib vaccine than girls. The observed gender differences in the AEFIs in our study and other parts of China may be explained by the son preference culture in China [33]. In traditional Chinese culture, boys are more valued than girls because they can continue the family line and care for their parents in old age [33]. Therefore, parents may pay more attention to boys’ health and are more likely to report AEFIs for boys. Alternatively, the gender differences in AEFIs may be related to the different immune responses between males and females, which requires further research [34]. Due to differences in factors such as genetics, sex hormones, and immune cell populations, males and females may have different susceptibility to AEFIs following the Hib vaccines [35]. For age distribution, AEFIs mainly occur in infants under 1 year old, which is in line with the existing Hib vaccination procedures in Zhejiang Province. Infants, especially those under the age of 1 year, have immature immune systems, which may lead to a higher risk of AEFIs [36].

For seasonal distribution, AEFIs were more common in summer and autumn, which may be explained by multiple environmental and behavioral factors. First, the hot, humid weather in summer and autumn may lead to more frequent skin reactions, such as dermatitis and rash. Second, children wear fewer clothes in these seasons, which makes it easier to detect general reactions, such as injection site redness, swelling, and induration, thus resulting in more reports. Third, hot weather can contribute to increased emotional distress due to physical discomfort, decreased appetite, sleep disturbances, and hormonal changes [37]. Therefore, children are more likely to develop anxiety, irritability, crying, and restlessness symptoms. For regional distribution, the reported incidence of Hib vaccine-related AEFIs varies greatly across the cities in Zhejiang Province, from 24.30/100,000 doses to 86.61/100,000 doses, which also echoes the finding in Hubei Province, showing a range of 10.06/100,000 doses to 132.65/100,000 doses across various cities [33]. The reporting disparities by region may be explained by the differences in administrative emphasis, staff training, and parental awareness in various cities. In addition, most AEFI cases occurred on the day after vaccination, highlighting the need for the close monitoring of adverse reactions following vaccination to guide timely management. In addition, pre-vaccination health inquiries should be strengthened, and vaccination should be delayed if symptoms such as a fever and cold occur to minimize the occurrence of incidental events.

In addition, the reported incidence rates of Hib vaccine-related AEFIs, including common and rare adverse reactions, were much higher in the booster immunization stage (fourth dose) than those in the basic immunization stage (first to third dose), which is consistent with other research results [38,39]. This may be because the levels of specific and non-specific antibodies in the body increase with the increasing number of vaccination doses. During the booster vaccination stage, the antigen enters the body and combines with the previous antibodies to form immune complex deposition, thereby triggering hypersensitivity reactions. This calls for special attention and the close monitoring of adverse reactions for booster doses. In addition, Zhejiang Province is a highly developed, large-population province in China; some infants and young children may be sent outside Zhejiang to be raised by grandparents after being born in Zhejiang for a period of time until they return to Zhejiang again before going to school. These children may receive the second and third doses of Hib vaccines outside Zhejiang, resulting in a decrease in the number of vaccines and vaccine-related AEFIs in Zhejiang. Compared with a vaccination of one vaccine alone, the incidence of reported adverse reactions, such as fever, is significantly increased when combined with multiple vaccines, which may be related to the increase in ingredients such as antigens, adjuvants, and preservatives [40]. The WHO suggests that the simultaneous vaccination of multiple vaccines will not have negative effects. The US CDC’s Immunization Practice Guidelines specify that the co-administration of several inactivated vaccines or an inactivated vaccine and a live attenuated vaccine at the same time without any time interval will lead to similar levels of seroconversion rates and adverse reaction rates to those when the vaccines are administered alone [41].

Our study has several strengths. First, it fills in the research gap by focusing on the safety profile of the more affordable Hib monovalent vaccines in China, rather than the Hib conjugate vaccines that are freely available via NIP in other countries. Second, the use of a large dataset that was collected through a national surveillance system (CNAEFIS) enhances the study’s generalizability, credibility, and reliability. Third, the graphic depiction of AEFI incidence across various regions provides an intuitive way to understand patterns, trends, relationships, and geographic context. Fourth, the comparison of AEFIs by a wide range of factors encompassing both individual (such as age and gender) and environmental levels (such as season and location) offers a comprehensive and nuanced understanding of the unique patterns and characteristics of AEFIs. Finally, this study effectively highlights the incidence and characteristics of AEFIs, contributing to an important public health discussion. The findings offer valuable epidemiological data for policymakers, particularly given China’s high burden of Hib-related diseases and its current lack of nationwide vaccine inclusion in the NIP.

Despite these strengths, our findings should be interpreted with caution due to several limitations. First, a major limitation of the passive surveillance system, CNAEFIS, is the potential under-reporting of AEFIs, as only authorized healthcare providers can submit the reports to the system. Healthcare providers may encounter various challenges in reporting AEFIs, including a lack of knowledge and awareness of the severity of the AEFIs and the importance of reporting, limited time, resources, and incentives for reporting AEFIs in busy clinical settings, as well as other logistic issues. Under-reporting may lead to the inaccurate assessment and incomplete understanding of the actual incidence and distribution of AEFIs in the population, which may delay or hinder timely and effective interventions [42]. Furthermore, under-reporting may cause limited resources to be misallocated to places with higher reporting rates, rather than places with higher needs but lower reporting rates [42]. To address the under-reporting in passive surveillance, we recommend the following strategies: (1) strengthening training, communication, and resources for healthcare providers to enhance their reporting awareness and quality, (2) offering incentives and positive feedback to motivate and encourage healthcare providers to actively report AEFIs, (3) streamlining the reporting system and procedure to ease work burden [43]. Additionally, incorporating other information sources, such as active and proactive surveillance systems, to obtain comprehensive data can also help overcome the limitation of under-reporting in passive surveillance [44].

Second, AEFIs are mainly reported by grassroots personnel, whose reporting capabilities may vary due to various levels of awareness, knowledge, experience, and training related to AEFIs. Third, the AEFI reporting quality varies greatly in different regions, which may affect the accuracy of the cross-city evaluation of AEFIs. Fourth, information about the Hib vaccine brands was not available due to the privacy protection of information for each vaccine manufacturer. Therefore, we were not able to compare the AEFIs of various vaccine brands produced by different companies, which may introduce a potential bias. Fifth, there is insufficient evidence of the causes for some AEFIs, especially severe reactions, and it is challenging to accurately identify the cause for AEFIs when multiple vaccines are administered at the same time. Future high-quality active surveillance systems and cohort studies are needed to address these limitations.

## 5. Conclusions and Recommendations

In conclusion, our study showed that the incidence rate of Hib vaccine-related AEFIs in Zhejiang Province from 2017–2023 was at a low level. Most of the AEFIs were common adverse reactions that manifested mainly as fever, injection site redness and swelling, and crying, which resolved spontaneously without treatment. The rates of rare and severe AEFIs were extremely low and did not cause any severe consequences. More AEFIs were reported among male participants under the age of one and when administration occurred in summer or autumn or during the booster immunity stage, indicating close monitoring for these populations and occasions. These results provide preliminary evidence of the safety profile of the Hib vaccine at a provincial level, which adds further support for its broader implementation in other provinces and at the national level.

While it is highly recommended to include the Hib vaccine in China’s NIP, several challenges remain to be addressed. First, the centralized funding mechanism for the NIP and the high costs of non-NIP vaccines may affect the quality and quantity of the Hib vaccine provision, hindering its inclusion in the NIP [45]. Second, a lack of attention to the life course and high-risk population may lead to the inadequate availability of vaccines targeted at certain populations, decreasing the effectiveness of the NIP [45]. Third, significant regional differences exist in the service capacity of vaccination (such as vaccination units and workforce) and standardized vaccination procedures, which may limit the successful expansion of the NIP [45]. Fourth, the prevalent vaccine hesitation in the public due to misconceptions about vaccine quality, safety, and efficacy may lead to weak demand-side drivers of vaccination, further impeding the NIP expansion [45]. To ensure the effectiveness and sustainability of the inclusion of the Hib vaccine in China’s NIP, multiple measures should be taken. These include increasing vaccination financing, encompassing the whole life course of individuals, overcoming regional disparities in immunization practices, improving the accessibility of vaccination services, strengthening the workforce, and enhancing information sharing and transparency to foster public trust [8,45].

Given the high disease burden of Hib and the significant public health implications of the Hib vaccination, future studies should adopt a more comprehensive evaluation framework to better inform multi-criteria decision-making regarding the inclusion of the Hib vaccine in China’s NIP [46]. First, large-scale, multi-center epidemiological studies are needed to not only investigate the disease burden and health impact of Hib but also evaluate the accessibility, affordability, and equity of the Hib vaccines [46]. Second, future research should conduct economic evaluations using robust modeling analyses to assess both the direct and indirect cost-effectiveness of the Hib vaccination to better inform decision-making [47]. Third, regional variations should be considered when investigating the disease burden and vaccine access, which can guide further targeted, individualized policymaking that best meets the needs of each region [48]. Fourth, public preference and engagement should also be emphasized to foster trust, ensure support, and improve the coverage of the Hib vaccine [49]. Finally, a comprehensive AEFI surveillance system that integrates both active and passive information sources is crucial to provide high-quality evidence for policymaking [50].

## Figures and Tables

**Table 1:** Reported Hib vaccine-related AEFIs in Zhejiang Province by year and category from 2017 to 2023.

Year	Doses (10,000)	Common Adverse Reaction		Rare Adverse Reaction		Coincidental Event		Total	
		No.	Incidence Rate * (95%CI)	No.	Incidence Rate * (95%CI)	No.	Incidence Rate * (95%CI)	No.	Incidence Rate * (95%CI)
2017	61.36	267	43.51 (38.60–49.05)	24	3.91 (2.63–5.82)	5	0.81 (0.35–1.91)	296	48.24 (43.05–54.05)
2018	75.41	383	50.79 (45.95–56.13)	49	6.50 (4.92–8.59)	7	0.93 (0.45–1.92)	439	58.21 (53.02–63.92)
2019	48.91	319	65.22 (58.45–72.78)	30	6.13 (4.30–8.76)	8	1.64 (0.83–3.23)	357	72.99 (65.81–80.96)
2020	29.41	221	75.14 (65.87–85.72)	20	6.80 (4.40–10.50)	4	1.36 (0.53–3.50)	245	83.30 (73.51–94.40)
2021	23.92	127	53.10 (44.63–63.16)	10	4.18 (2.27–7.70)	0	0.00 (0.00–1.61)	137	57.28 (48.46–67.70)
2022	19.92	122	61.24 (51.30–73.11)	1	0.50 (0.09–2.84)	0	0.00 (0.00–1.93)	123	61.74 (51.75–73.65)
2023	17.23	138	80.09 (67.80–94.61)	5	2.90 (1.24–6.79)	0	0.00 (0.00–2.23)	143	82.99 (70.46–97.75)
Total	276.16	1577	57.10 (54.35–59.99)	139	5.03 (4.26–5.94)	24	0.87 (0.58–1.29)	1740	63.01 (60.12–66.04)

* The incidence rate (/100,000) was calculated as the number of AEFI reports/administered doses × 100,000. Hib: *Haemophilus influenzae* type b; AEFIs: adverse events following immunization; incidence rate was reported as /100,000 doses.

**Table 2:** Distribution of clinical symptoms of common and rare adverse reactions after Hib vaccination in Zhejiang Province from 2017 to 2023.

Symptoms	Number	Proportion (%) ^#^	Incidence Rate (/100,000 Doses) * (95%CI)
Common adverse reactions (n = 1577)			
Fever	919	52.82	33.28 (31.19–35.50)
37.1–37.5	52	2.99	1.88 (1.44–2.47)
37.6–38.5	315	18.10	11.41 (10.21–12.74)
≥38.6	552	31.72	19.99 (18.39–21.73)
Injection site redness and swelling	684	39.31	24.77 (22.98–26.69)
≤2.5	122	7.01	4.42 (3.70–5.27)
2.5–5	390	22.41	14.12 (12.79–15.59)
≥5	172	9.89	6.23 (5.36–7.23)
Crying	396	22.76	14.34 (12.99–15.82)
Induration	273	15.69	9.89 (8.78–11.13)
≤2.5	109	6.26	3.95 (3.27–4.76)
2.5–5	123	7.07	4.45 (3.73–5.31)
≥5	41	2.36	1.48 (1.09–2.01)
Rash	129	7.41	4.67 (3.93–5.55)
Loss of appetite	94	5.40	3.40 (2.78–4.16)
Itching	78	4.48	2.82 (2.26–3.52)
Weakness	64	3.68	2.32 (1.82–2.96)
Lethargy	36	2.07	1.30 (0.94–1.80)
Vomit	35	2.01	1.27 (0.91–1.76)
Myalgia	28	1.61	1.01 (0.70–1.47)
Abdominal pain and diarrhea	24	1.38	0.87 (0.58–1.29)
Cough	11	0.63	0.40 (0.22–0.71)
Laryngeal redness	10	0.57	0.36 (0.20–0.67)
Nausea	7	0.40	0.25 (0.12–0.52)
Sweating	7	0.40	0.25 (0.12–0.52)
Runny nose	4	0.23	0.14 (0.06–0.37)
Paleness	2	0.11	0.07 (0.02–0.26)
Other	21	1.21	0.76 (0.50–1.16)
Rare adverse reactions (n = 139)			
Allergic rash	83	27.33	3.01 (2.42–3.73)
Urticaria	27	27.00	0.98 (0.67–1.42)
Maculopapular rash	14	10.07	0.51 (0.30–0.85)
Angioedema	3	2.16	0.11 (0.04–0.32)
Febrile convulsions	3	2.16	0.11 (0.04–0.32)
Lymphadenitis and lymphangitis	2	1.44	0.07 (0.02–0.26)
Thrombocytopenic purpura	2	1.44	0.07 (0.02–0.26)
Acute allergic reactions	1	0.72	0.04 (0.01–0.21)
Erythema multiforme	1	0.72	0.04 (0.01–0.21)
Other	3	2.16	0.11 (0.04–0.32)

^#^ The proportion was calculated as the number of reported cases of the symptom/the total number of reported cases (1740). * The incidence rate (/100,000) was calculated as the number of AEFI reports/administered doses × 100,000. Hib: *Haemophilus influenzae* type b.

**Table 3:** Epidemiological distribution of AEFIs following Hib vaccination from 2017 to 2023 in Zhejiang Province.

Variables		AEFI Cases		Incidence Rate (/100,000 Doses) * (95%CI)	*p*
		No.	Proportion (%) ^#^		
Gender					*χ*^2^ = 16.8572, *p* ≤ 0.0001
	Male	993	57.07	68.94 (64.79–73.37)	
	Female	747	42.93	56.53 (52.62–60.73)	
Age (years)					*χ*^2^ = 45.0140, *p* ≤ 0.0001
	0	808	46.44	54.78 (51.13–58.69)	
	1	770	44.25	69.87 (65.11–74.98)	
	2	120	6.90	87.03 (72.79–104.04)	
	3	30	1.72	105.85 (74.16–151.07)	
	≥4	12	0.69	65.36 (37.39–114.22)	
Season					*χ*^2^ = 355.1184, *p* ≤ 0.0001
	Spring	276	15.86	43.92 (39.04–49.42)	
	Summer	677	38.91	86.13 (79.88–92.86)	
	Autumn	560	32.18	73.51 (67.67–79.85)	
	Winter	227	13.05	38.78 (34.05–44.16)	

^#^ The proportion was calculated as the number of reported cases of the symptom/the total number of reported cases (1740). * The incidence rate (/100,000) was calculated as the number of AEFI reports/administered doses × 100,000. Hib: *Haemophilus influenzae* type b; AEFIs: adverse events following immunization.

**Table 4:** Hib vaccine-related AEFIs by location from 2017 to 2023 in Zhejiang Province.

City	AEFI		Rare Adverse Reactions	
	No.	Incidence Rate * (/100,000 Doses) (95%CI)	No.	Incidence Rate * (/100,000 Doses) (95%CI)
Hangzhou	297	81.29 (72.56–91.07)	22	6.02 (3.98–9.12)
Wenzhou	253	54.67 (48.34–61.84)	19	4.11 (2.63–6.41)
Jinhua	228	60.05 (52.75–68.36)	9	2.37 (1.25–4.51)
Taizhou	212	66.62 (58.24–76.21)	17	5.34 (3.34–8.56)
Ningbo	208	53.57 (46.77–61.36)	6	1.55 (0.71–3.37)
Jiaxing	167	86.61 (74.44–100.77)	16	8.30 (5.11–13.48)
Shaoxing	134	54.83 (46.30–64.93)	21	8.59 (5.62–13.14)
Huzhou	103	65.01 (53.61–78.83)	16	10.10 (6.22–16.41)
Quzhou	71	60.51 (47.98–76.31)	12	10.23 (5.85–17.88)
Lishui	63	53.41 (41.75–68.32)	1	0.85 (0.15–4.80)
Zhoushan	4	24.30 (9.45–62.47)	0	0.00 (0.00–23.33)
Total	1740	63.01 (60.12–66.04)	139	5.03 (4.26–5.94)

* The incidence rate (/100,000) was calculated as the number of AEFI reports/administered doses × 100,000. Hib: *Haemophilus influenzae* type b; AEFIs: adverse events following immunization.

**Table 5:** Hib vaccine-related AEFIs by doses from 2017 to 2023 in Zhejiang Province.

Doses	AEFI		Common Adverse Reactions		Rare Adverse Reactions	
	No.	Incidence Rate * (95%CI)	No.	Incidence Rate * (95%CI)	No.	Incidence Rate * (95%CI)
1st	732	59.36 (55.2–163.82)	671	54.41 (50.45–58.69)	49	3.97 (3.01–5.25)
2nd	278	45.37 (40.35–51.03)	250	40.80 (36.05–46.18)	25	4.08 (2.76–6.02)
3rd	285	55.37 (49.31–62.18)	247	47.99 (42.37–54.36)	36	6.99 (5.05–9.68)
4th	445	110.94 (101.10–121.73)	409	101.96 (92.55–112.33)	29	7.23 (5.03–10.38)

* The incidence rate (/100,000) was calculated as the number of AEFI reports/administered doses × 100,000. Hib: *Haemophilus influenzae* type b; AEFIs: adverse events following immunization.

**Table 6:** Hib vaccine-related AEFIs by co-administration status from 2017 to 2023 in Zhejiang Province.

Co-Administration Status	Total AEFIs No.	Fever		Redness and Swelling		Crying	
		No.	% ^#^	No.	% ^#^	No.	% ^#^
No co-administration	1465	723	49.35	630	43.00	323	22.05
Co-administration with one vaccine (HIB + X) ^&^	257	185	71.98	51	19.84	67	26.07
Co-administration with two vaccines (HIB + X1 + X2) ^&^	18	11	61.11	3	16.67	6	33.33
	1740	*χ*^2^ = 44.6467, *p* < 0.0001		*χ*^2^ = 52.9916, *p* < 0.0001		*χ*^2^ = 2.6644, *p* = 0.1026	

^#^ The proportion was calculated as the number of reported cases in each category/the total number of reported cases. ^&^ Common vaccines that are given alongside the HIB vaccine include the diphtheria–tetanus vaccine, EV71 vaccine, inactivated polio vaccine, etc.

**Table 7:** Details of six severe cases of Hib vaccine-related AEFIs.

No.	Age	Gender	Vaccination Time	Doses	AEFI Occurrence Time	Co-Administration	Clinical Diagnosis	Classification
1	1	M	24 February 2020	4	24 February 2020	DPT	Febrile convulsions	Rare adverse reactions
2	1	F	10 March 2020	1	10 March 2020		Febrile convulsions	Rare adverse reactions
3	1	M	20 March 2017	1	20 March 2017		Febrile convulsions	Rare adverse reactions
4	0	M	12 March 2018	1	12 March 2018		Thrombocytopenic purpura	Rare adverse reactions
5	0	F	26 November 2019	1	28 November 2019		Thrombocytopenic purpura	Rare adverse reactions
6	0	F	9 March 2017	1	2 April 2017		Allergic purpura	Coincidental event

Hib: *Haemophilus influenzae* type b; AEFIs: adverse events following immunization.

## Data Availability

Data cannot be shared openly due to the protection of patient information and privacy. Access to the data is subject to approval and a data-sharing agreement with Zhejiang CDC.
